# *Fusarium* Mycotoxins Disrupt the Barrier and Induce IL-6 Release in a Human Placental Epithelium Cell Line

**DOI:** 10.3390/toxins11110665

**Published:** 2019-11-14

**Authors:** Negisa Seyed Toutounchi, Astrid Hogenkamp, Soheil Varasteh, Belinda van’t Land, Johan Garssen, Aletta D. Kraneveld, Gert Folkerts, Saskia Braber

**Affiliations:** 1 Division of Pharmacology, Utrecht Institute for Pharmaceutical Sciences, Faculty of Science, Utrecht University, 3584 CG Utrecht, The Netherlands; s.seyedtoutounchi@uu.nl (N.S.T.); a.hogenkamp@uu.nl (A.H.); s.varasteh@uu.nl (S.V.); j.garssen@uu.nl (J.G.); A.D.Kraneveld@uu.nl (A.D.K.); g.folkerts@uu.nl (G.F.); 2 Department of Immunology, Danone Nutricia Research, 3584 CT Utrecht, The Netherlands; Belinda.vantland@danone.com; 3 Laboratory of Translational Immunology, Wilhelmina Children’s Hospital, University Medical Center Utrecht, 3584 CX Utrecht, The Netherlands; 4 Veterinary Pharmacology & Therapeutics, Institute of Risk Assessment Sciences, Faculty of Veterinary Medicine, Utrecht University, 3584 CL Utrecht, The Netherlands

**Keywords:** deoxynivalenol, zearalenone, T-2 toxin, mycotoxins, placenta, tight junction, inflammation, BeWo cells, barrier function

## Abstract

Deoxynivalenol, T-2 toxin, and zearalenone, major *Fusarium* mycotoxins, contaminate human food on a global level. Exposure to these mycotoxins during pregnancy can lead to abnormalities in neonatal development. Therefore, the aim of this study was to investigate the effects of *Fusarium* mycotoxins on human placental epithelial cells. As an in vitro model of placental barrier, BeWo cells were exposed to different concentrations of deoxynivalenol, zearalenone or T-2 toxin. Cytotoxicity, effects on barrier integrity, paracellular permeability along with mRNA and protein expression and localization of junctional proteins after exposure were evaluated. Induction of proinflammatory responses was determined by measuring cytokine production. Increasing mycotoxin concentrations affect BeWo cell viability, and T-2 toxin was more toxic compared to other mycotoxins. Deoxynivalenol and T-2 toxin caused significant barrier disruption, altered protein and mRNA expression of junctional proteins, and induced irregular cellular distribution. Although the effects of zearalenone on barrier integrity were less prominent, all tested mycotoxins were able to induce inflammation as measured by IL-6 release. Overall, *Fusarium* mycotoxins disrupt the barrier of BeWo cells by altering the expression and structure of junctional proteins and trigger proinflammatory responses. These changes in placental barrier may disturb the maternal–fetal interaction and adversely affect fetal development.

## 1. Introduction

Mycotoxins are naturally produced as secondary metabolites of fungi species and contaminate a wide range of foods, especially cereal and grain products [1]. *Fusarium* species are one of the most prevalent contaminants of cereal grains. Deoxynivalenol (DON), T-2 toxin, and zearalenone (ZEN) are the major *Fusarium* mycotoxins occurring in human food worldwide [2]. Despite all efforts and legislations to limit the fungal contamination, human exposure to mycotoxins cannot be completely prevented, as they are natural contaminants of agricultural products, and the invasion of toxigenic fungi species occurs regularly in food supplies at a global level [3]. Moreover, masked or metabolized mycotoxins are of great concern in risk assessments, because they are hardly detected by conventional analytical methods and can transform to parent mycotoxins after ingestion [4]. Due to the variation in the mycotoxin content of human foods and seasonal differences in fungal infection, it is difficult to make an exact estimation of human exposure levels. However, investigating the occurrence of *Fusarium* mycotoxins and their modified forms in cereal grains originating from different European countries shows a considerable level of contamination exceeding the maximum tolerable daily intake (TDI) in some cases [5].

As exposure is inevitable, it seems essential to explore the health effects of different mycotoxins in humans, especially high-risk groups, including fetuses, neonates, and infants [6]. 

Several studies have demonstrated that DON, ZEN, and T-2 toxin can pass through the placenta [7,8,9] and adversely affect the development of the fetus during pregnancy. Deoxynivalenol, ZEN, and their major metabolites are detected in fetal and placental samples of pregnant sows and rats receiving contaminated diets during pregnancy [8,10,11]. Deoxynivalenol exposure in pregnant mice can lead to increased resorption rate, structural and functional damages in placenta, and fetal skeletal malformations [12,13]. Prenatal exposure to ZEN, as a non-steroidal estrogenic mycotoxin, can cause long-term adverse effects on the reproductive system of the first generation female offspring (F1), including abnormal and unfunctional ovarian follicles [14]. Studies have shown that ZEN can inhibit the mRNA expression of the estrogen receptor α (Esr1) and alter the expression of major ABC transporters in placentae collected from pregnant rats and in BeWo cells in vitro, indicating that this mycotoxin can alter placental transportation and, consequently, increase the risk of exposure to different xenobiotics and toxins in fetuses [14,15,16]. Studies have shown that T-2 toxin can also pass through the placenta and distribute to fetal tissues [9] which can induce fetal death, brain damage [17,18], skeletal malformation [19], thymus atrophy [20], and suppression of humoral immunity [21]. 

During pregnancy, the placenta is the only link between the fetus and mother, mediating the maternal–fetal transfer of nutrients and metabolic waste products while also secreting hormones that maintain pregnancy [22]. The placenta is also important as a barrier against pathogens and paracellular diffusion of chemicals and toxins [23]. The epithelial layer of the placenta consists of cytotrophoblasts and syncytiotrophoblasts and controls maternal–fetal transfer [24]. Therefore, any disruption of the integrity of this layer can lead to imbalanced maternal–fetal transportation of nutrients and hazardous chemicals. In the placental epithelium, junctional proteins—including tight junctions (TJs) and adherent junctions (AJs)—are responsible for the preservation of this barrier. These proteins maintain the integrity and control paracellular transport of macromolecules across the placental epithelium [25,26,27]. Pathophysiological conditions that trigger the inflammatory responses in placenta can result in disassembly of junctional proteins at trophoblast cells and weaken the integrity, leading to detrimental consequences for the development of the fetus [28]. 

There are no reports on the effects of *Fusarium* mycotoxins on the placental barrier function. Therefore, in this study, the effects of major *Fusarium* mycotoxins on the placental barrier function were investigated by evaluating the expression and localization of junctional proteins. In addition, cell viability and inflammatory responses in placental epithelium were determined after mycotoxin exposure. To this end, BeWo cells were used as an in vitro model for placental epithelium [22]. This cell line is derived from human choriocarcinoma which retain cell properties similar to human trophoblasts which are in direct contact with maternal blood supply [29]; therefore, they serves as a suitable in vitro model of the rate-limiting barrier to maternal–fetal exchange [23]. Untreated BeWo cells show the morphological and biochemical characterizations of undifferentiated cytotrophoblasts [30] and, therefore, can be a representative of placental epithelium of the first trimester of pregnancy. 

## 2. Results

### 2.1. Concentrations of 8 µM DON, 16 µM ZEN, and 8 nM T-2 Toxin are Cytotoxic for BeWo Cells

**Figure 1:**
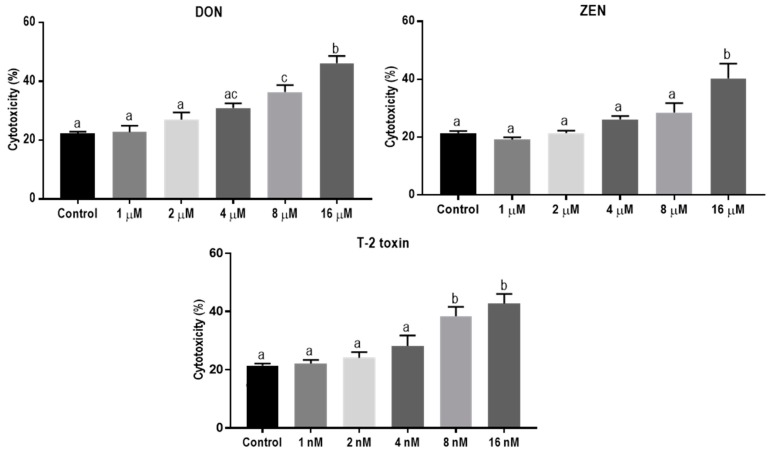
Lactate dehydrogenase (LDH) release in BeWo cells incubated for 24 h with increasing concentrations of deoxynivalenol (DON), zearalenone (ZEN) and T-2 toxin. Data are representative of three independent experiments, each performed in triplicate, and expressed as the mean ± SEM. Different lowercase letters denote significant differences among the treatments (*p* < 0.05).

The direct cytotoxic effects of DON, ZEN, and T-2 toxin on BeWo cells were assessed by measuring lactate dehydrogenase (LDH) release in cell supernatants after 24 h exposure. The LDH levels increased after exposure to increasing concentrations of DON, ZEN or T-2 toxin. Concentrations of 8 µM, 16 µM, and 8 nM of DON, ZEN, and T-2 toxin, respectively, led to significantly higher LDH release compared to the control (Figure 1). In all subsequent experiments, concentrations equal to or below these toxic levels of mycotoxins were used.

### 2.2. Mycotoxins Exposure Disrupts the Integrity and Increases Permeability of the BeWo Cell Layer

**Figure 2:**
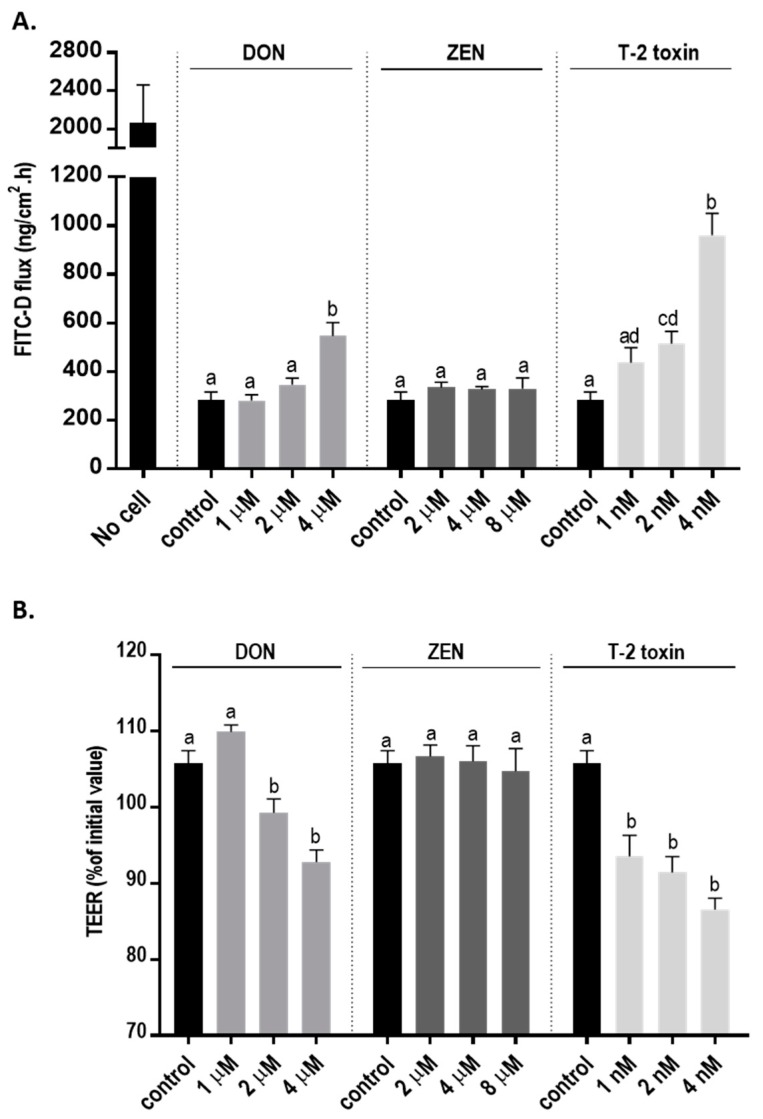
Effects of deoxynivalenol (DON), T-2 toxin, and zearalenone (ZEN) exposure on the integrity of the BeWo cell monolayer. The changes in (**A**) transepithelial electrical resistance (TEER) and (**B**) fluorescein isothiocyanate-dextran (FITC-D) flux from apical to basolateral compartment after 24 h exposure to mycotoxins at the apical side are shown. Data are expressed as the mean ± SEM of three independent experiments, each performed in triplicate. Different lowercase letters denote significant differences among groups. (*p* < 0.05).

The integrity of the cellular monolayer after mycotoxins exposure was evaluated by adding increasing concentration of mycotoxins to the apical compartments of Transwell inserts. Transepithelial electrical resistance (TEER) was significantly decreased after 24 h exposure to DON and T-2 toxin, while ZEN had no effect on TEER values (Figure 2A). Correspondingly, a significant increase in paracellular transport of fluorescein isothiocyanate-dextran (FITC-D) across the cell monolayer from the apical to the basolateral chamber after DON and T-2 toxin exposure was observed; but, after ZEN exposure, the paracellular transport of FITC-D was unaffected (Figure 2B).

### 2.3. Mycotoxins Exposure Alters Gene Expression of Junctional Proteins

**Figure 3:**
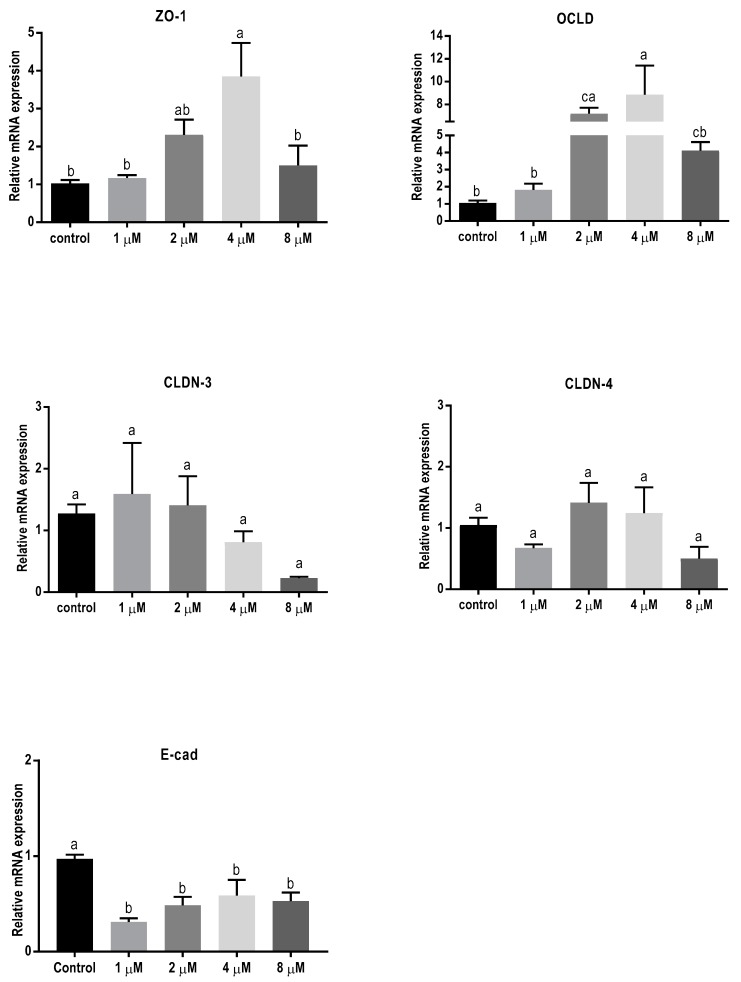
Effects of deoxynivalenol (DON) exposure on mRNA levels of junctional proteins in BeWo cells. The mRNA expression of occludin (OCLD), zonula occludens protein-1 (ZO-1), claudin (CLDN)-3 and 4, and E-cadherin (E-cad) in BeWo cells after 24 h exposure to different concentrations of DON. Data are expressed as the mean ± SEM of three independent experiments, each performed in triplicate. Different lowercase letters denote significant differences among groups (*p* < 0.05).

In order to assess the effect of 24 h exposure to different mycotoxins on the barrier function of BeWo cells in more detail, the mRNA expression levels of different junctional proteins, including occludin (OCLD), zonula occludens protein-1 (ZO-1), claudin (CLDN)-3 and 4, and E-cadherin, were assessed by real-time polymerase chain reaction (qPCR) analysis. Exposure to the low concentrations of DON (2 and 4 µM) resulted in increased mRNA levels of ZO-1 and OCLD, but no significant effect on CLDN-3 and CLDN-4 mRNA levels were observed (Figure 3). All DON concentrations significantly decreased the mRNA expression of E-cadherin. 

**Figure 4:**
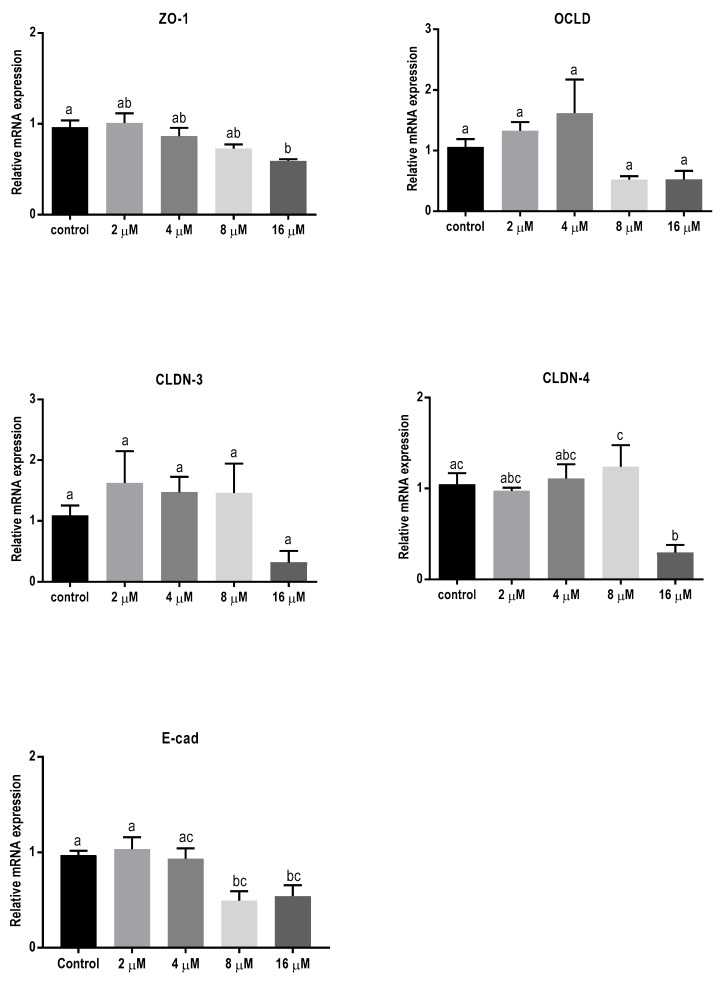
Effects of zearalenone (ZEN) exposure on mRNA levels of junctional proteins in BeWo cells. The mRNA expression of occludin (OCLD), zonula occludens protein-1 (ZO-1), claudin (CLDN)-3 and 4, and E-cadherin (E-cad) in BeWo cells after 24 h exposure to different concentrations of ZEN. Data are expressed as the mean ± SEM of three independent experiments, each performed in triplicate. Different lowercase letters denote significant differences among groups (*p* < 0.05).

The lower concentrations of ZEN showed no significant effect on mRNA level of all tight junctions (TJ) and E-cadherin (Figure 4), but the higher concentrations caused a reduction in mRNA levels of ZO-1, CLDN-4, and E-cadherin. 

**Figure 5:**
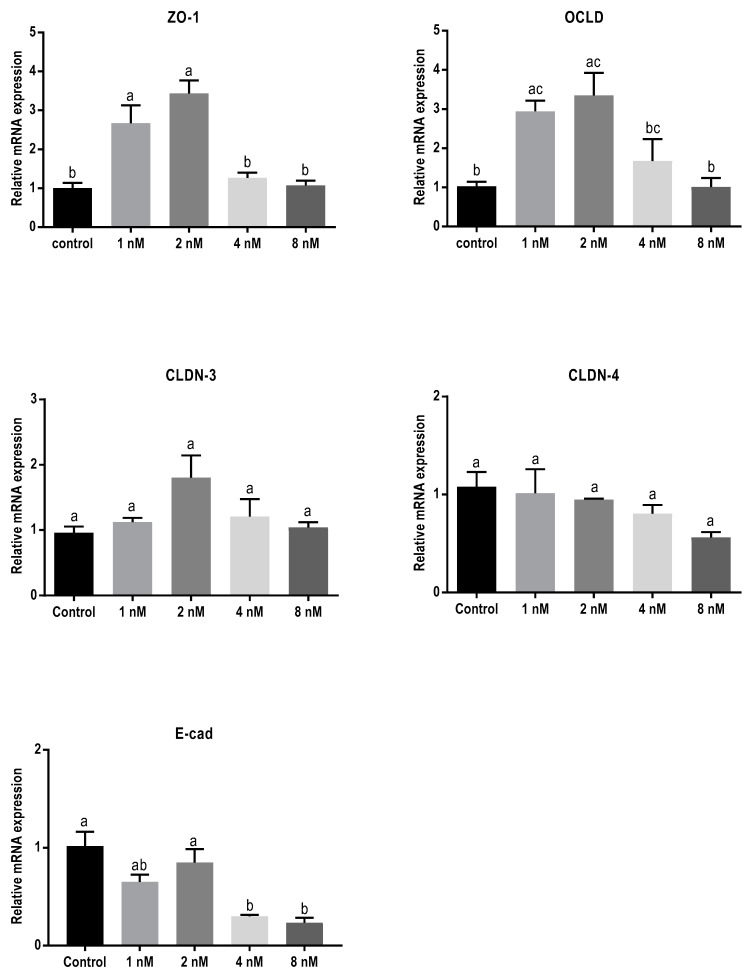
Effects of T-2 toxin exposure on mRNA levels of junctional proteins in BeWo cells. The mRNA expression of occludin (OCLD), zonula occludens protein-1 (ZO-1), claudin (CLDN)-3 and 4, and E-cadherin (E-cad) in BeWo cells after 24 h exposure to different concentrations of T-2 toxin. Data are expressed as the mean ± SEM of three independent experiments, each performed in triplicate. Different lowercase letters denote significant differences among groups (*p* < 0.05).

Exposure to T-2 toxin resulted in a similar mRNA-expression pattern as observed for cells exposed to DON (Figure 5). Concentrations of 1 and 2 nM of T-2 toxin significantly increased mRNA levels of ZO-1 and OCLD but had no significant effect on CLDNs. The lower concentrations of T-2 toxin had no effect on E-cadherin mRNA expression, but the higher concentrations caused significant decrease. 

### 2.4. Mycotoxins Exposure Modulates the Protein Expression of Junctional Proteins

**Figure 6:**
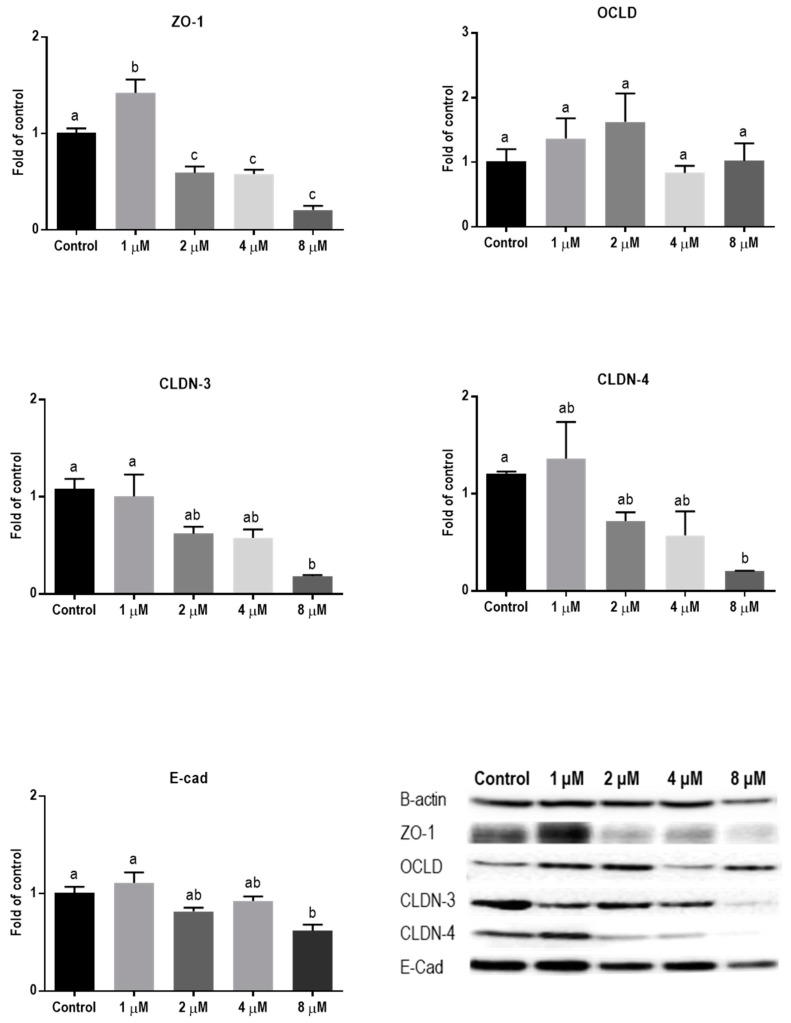
Effects of deoxynivalenol (DON) exposure on junctional proteins in BeWo cells. The protein expression of occludin (OCLD), zonula occludens protein-1 (ZO-1), claudin (CLDN)-3 and 4, and E-cadherin (E-cad) in BeWo cells after 24 h exposure to different concentrations of DON. Data are expressed as the mean ± SEM of three independent experiments, each performed in triplicate. Different lowercase letters denote significant differences among groups (*p* < 0.05).

**Figure 7:**
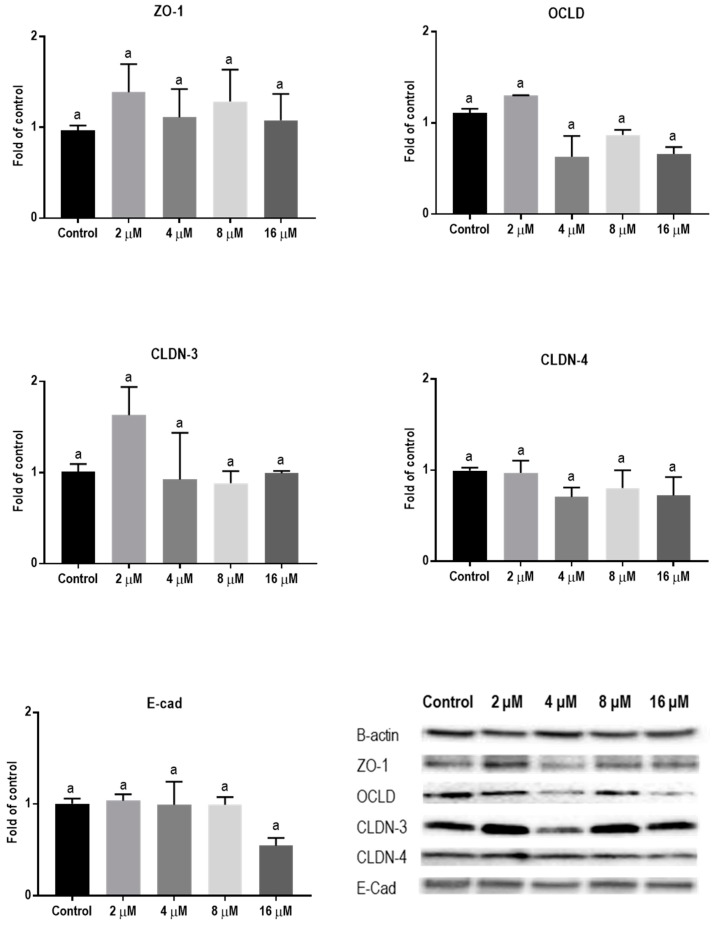
Effects of zearalenone (ZEN) exposure on junctional proteins in BeWo cells. The protein expression of occludin (OCLD), zonula occludens protein-1 (ZO-1), claudin (CLDN)-3 and 4, and E-cadherin (E-cad) in BeWo cells after 24 h exposure to different concentrations of ZEN. Data are expressed as the mean ± SEM of three independent experiments, each performed in triplicate. Different lowercase letters denote significant differences among groups (*p* < 0.05).

**Figure 8:**
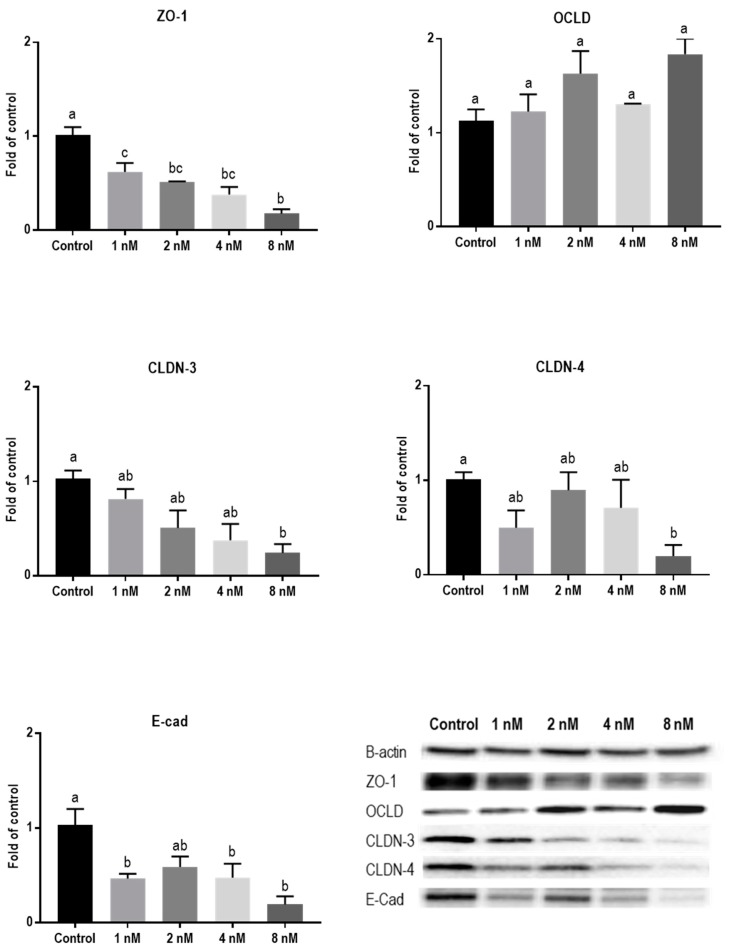
Effects of T-2 toxin exposure on junctional proteins in BeWo cells. The protein expression of occludin (OCLD), zonula occludens protein-1 (ZO-1), claudin (CLDN)-3 and 4, and E-cadherin (E-cad) in BeWo cells after 24 h exposure to different concentrations of T-2 toxin. Data are expressed as the mean ± SEM of three independent experiments, each performed in triplicate. Different lowercase letters denote significant differences among groups (*p* < 0.05).

Protein levels of TJs and E-cadherin in mycotoxin-exposed BeWo cells were assessed with Western blot analysis. The BeWo cells were collected after 24 h exposure to increasing concentrations of DON, ZEN, and T-2 toxin. The lower concentrations of DON increased the protein level of ZO-1 and had no effect on CLDN-3, CLDN-4, and E-cadherin, but higher concentrations caused significant reduction in expression of ZO-1, CLDNs, and E-cadherin proteins. The expression of OCLD was not significantly affected by DON (Figure 6). As shown in Figure 7, the effect of ZEN on the protein levels of TJs was not clear and only the higher concentration of ZEN decreased the expression of OCLD and E-cadherin, but these effects were not statistically significant. Expression of ZO-1 and E-cadherin were significantly reduced after exposure to T-2 toxin. The CLDN-3 and 4 protein levels were only significantly reduced by the higher concentration of 8 nM, and the expression of OCLD protein was not significantly affected (Figure 8). 

### 2.5. Mycotoxins Exposure Alters the Localization of Junctional Proteins

**Figure 9:**
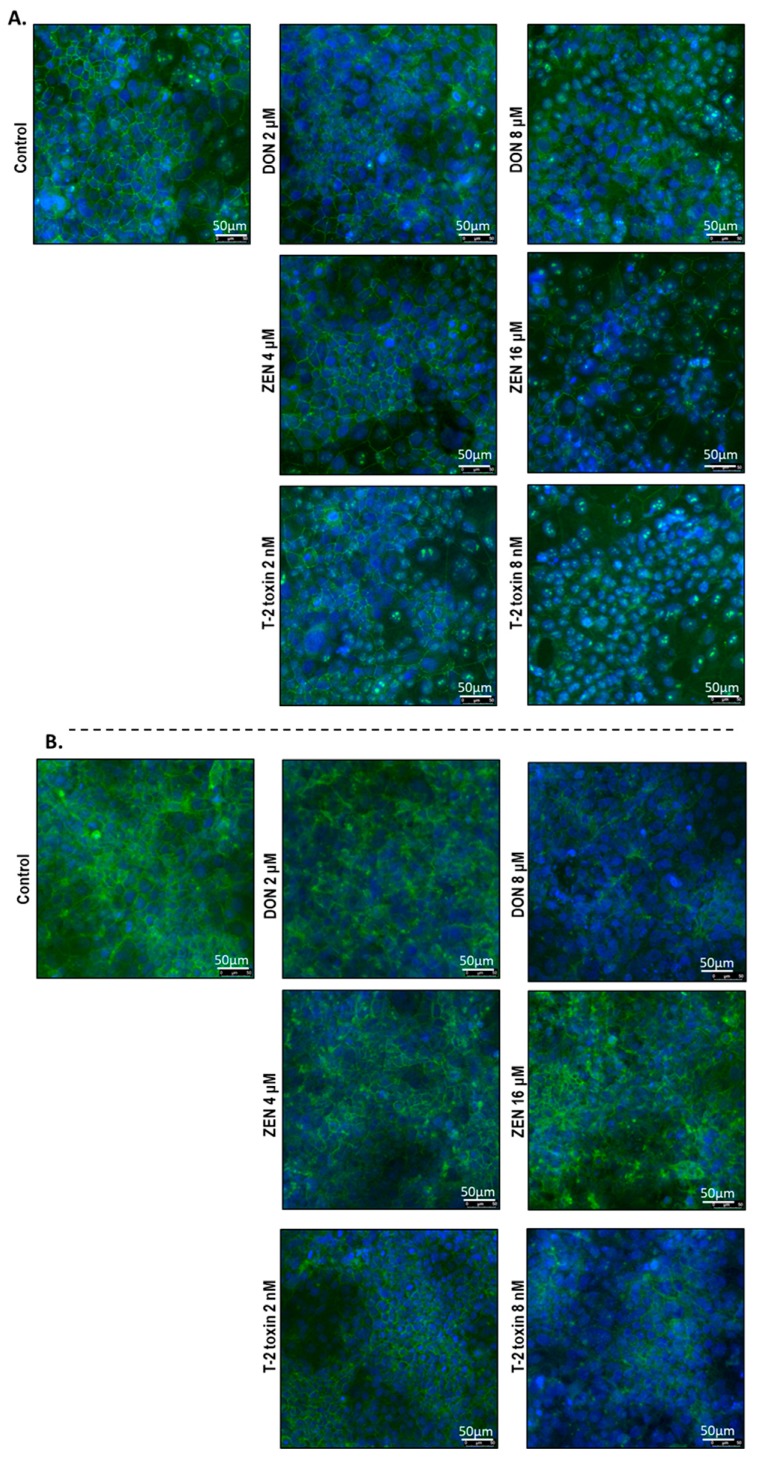
Effects of deoxynivalenol (DON), T-2 toxin, and zearalenone (ZEN) exposure on (**A**) occludin (OCLD) and (**B**) claudin (CLDN)-4 localization in BeWo cells, visualized by immunofluorescence staining (400× magnification). Scale bars represent 50 μm.

**Figure 10:**
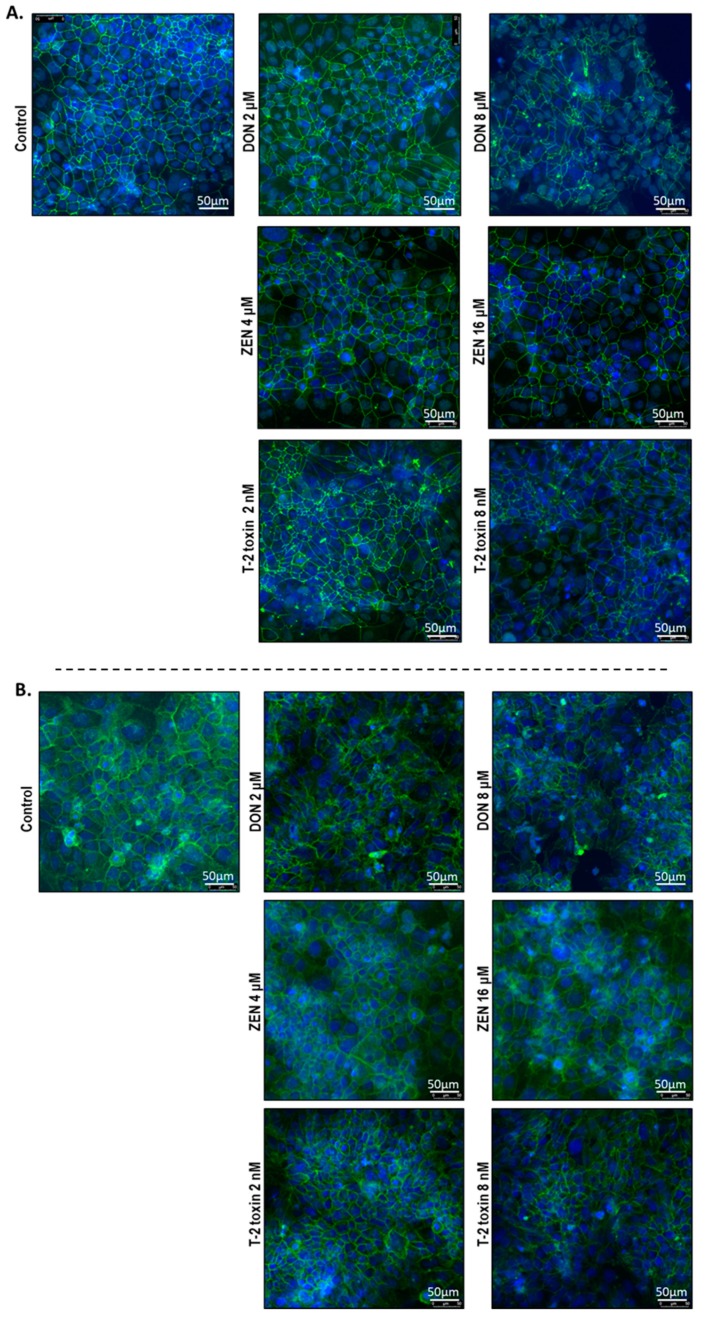
Effects of deoxynivalenol (DON), T-2 toxin, and zearalenone (ZEN) exposure on (**A**) occludens protein-1 (ZO-1) and (**B**) E-cadherin localization in BeWo cells, visualized by immunofluorescence staining (400× magnification). Scale bars represent 50 μm.

To investigate the cellular localization of junction proteins, immunofluorescence staining was performed using BeWo cells grown on 24-well plates incubated with or without increasing concentrations of DON, ZEN, or T-2 toxin for 24 h. In the intact BeWo cells OCLD, CLDN-4, ZO-1, and E-cadherin were localized at the cell membrane and formed continuous belt-like structures (Figure 9 and Figure 10). The DON and T-2 toxin exposure increased delocalization and intracellular accumulation of OCLD (Figure 9A) and decreased expression and induced a disturbed and irregular cellular distribution of CLDN-4 protein on the cell surface, especially by the higher concentrations (Figure 9B). The higher concentration of ZEN (16 μM) also caused similar modifications on CLDN-4 and OCLD, but the effect was less intense. The higher DON and T-2 toxin concentrations caused an obvious irregular assembly of E-cad and ZO-1 proteins, depicted as discontinuous belt-like structures around the cells. The ZEN exposure showed no visible effect on these two proteins (Figure 10A,B). 

### 2.6. Mycotoxins Exposure Increases Interleukin-6 (IL-6) mRNA Expression and Protein Release 

**Figure 11:**
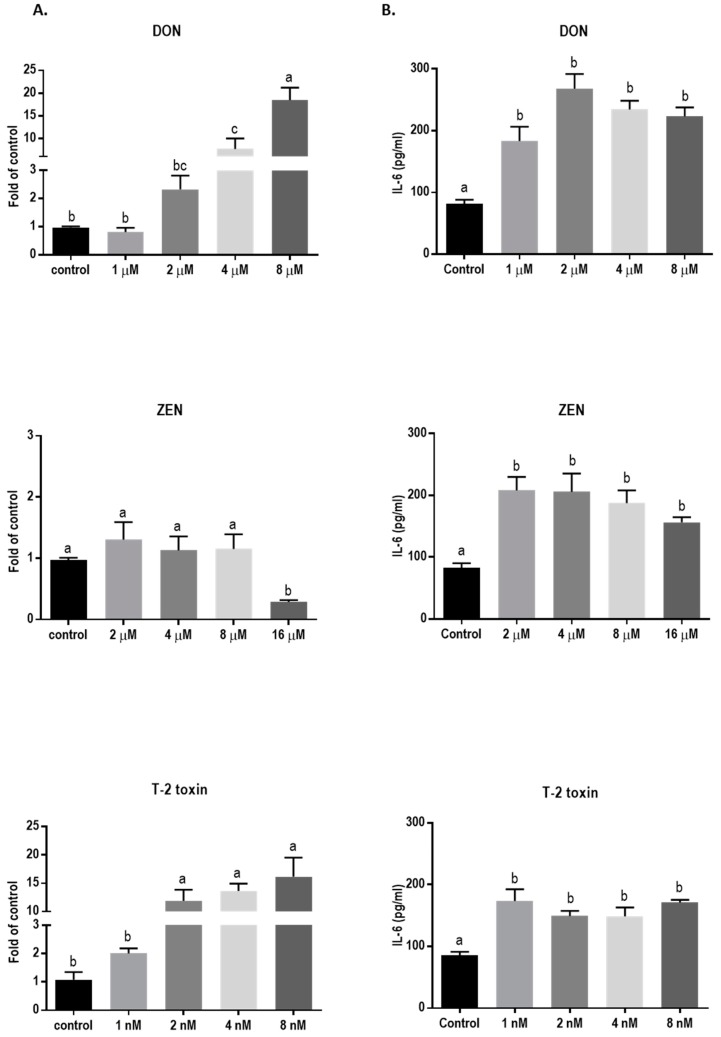
Effects of mycotoxin exposure on IL-6 mRNA expression and IL-6 secretion in BeWo cells. BeWo cells were incubated for 24 h with increasing concentrations of deoxynivalenol (DON), zearalenone (ZEN), and T-2 toxin and the IL-6 mRNA expression (**A**) and secretion (**B**) were measured. Data are expressed as the mean ± SEM of three independent experiments, each performed in triplicate. Different lowercase letters denote significant differences among groups (*p* < 0.05).

Exposure to DON and T-2 toxin caused a significant increase in the mRNA level of IL-6 (Figure 11A) and led to significantly higher levels of this cytokine in the cell supernatant compared to the untreated cells (Figure 11B). Although ZEN had no significant effect on the mRNA expression of IL-6, the cytokine release was increased, even with the lower concentrations of ZEN. However, none of the tested mycotoxins stimulated IL-8 and IL-1β cytokine release or mRNA expression (data not shown).

## 3. Discussion 

The placental epithelium covers the maternal surface of the placenta and forms a polarized layer of cells, where the microvillous membrane of the syncytiotrophoblasts is in direct contact with the maternal blood, while the basal membrane faces the fetal circulation [31]. This cell layer forms a semipermeable barrier by restricting the paracellular space with junctional proteins [27,32,33]. The placenta is essential for protecting and nourishing the fetus during pregnancy and has profound consequences for life-long health. Disruption of junctional proteins in placenta as a result of maternal exposure to toxic dietary contaminants can lead to impaired maternal–fetal barrier and endanger the growth and health of the fetus. 

Mycotoxins are natural food and feed contaminants which can significantly impact human and animal health. Limited studies report on the effects of mycotoxins on placental epithelium, therefore, the current study focused on the direct effect of exposure to three major *Fusarium* mycotoxins on barrier and immune function of human placental cells. There is scarce information about the exact serum levels of mycotoxins in different populations [34,35]; however, these dietary contaminants can be present in a wide range of food products, and the amount and the type of daily food intake varies for every individual. Therefore, in order to set proper limitations on mycotoxin exposure levels during pregnancy, it is important to determine the minimum concentrations that can impose toxic effects on human placental cells.

Exposure to increasing concentrations of mycotoxins for 24 h caused concentration-dependent cell toxicity in BeWo cells. The T-2 toxin exposure caused cellular leakage at much lower concentrations in comparison to the other toxins, whereas ZEN could be ranked as the least toxic mycotoxin among the three. The concentration of T-2 toxin in naturally contaminated grains and cereals and the average daily exposure to this toxin are usually lower than other *Fusarium* mycotoxins [36]. However, due to the fact of its high toxicity, it has a very low TDI level in humans compared to DON and ZEN (1, 0.25, and 0.02 µg/kg body weight/day for DON, ZEN, and T-2 toxin, respectively) [37,38,39,40], and based on the data from this study, exposure to very low concentrations of T-2 toxin can cause significant toxicity on BeWo cells. In this study, exposure to individual *Fusarium* mycotoxins was investigated. However, simultaneous exposure to different mycotoxins, which occurs naturally as a result of either contemporary production of different toxins from some fungal species or mixed fungi infections in commodities, is raising concerns about the possible additive or synergistic toxic effects [41]. The co-occurrence of low doses of different mycotoxins in food—especially the trichothecenes—may be even more toxic than predicted for the individual mycotoxins [42]. This underlines the importance of studying the effects of these toxins on vulnerable tissues such as the placenta.

The significant decrease in electrical resistance and increase in intracellular permeability of FITC-D after exposure to DON and T-2 toxin indicates the detrimental effect of these two mycotoxins on barrier function and integrity of placental epithelial cell line. Previous studies reported no effect of T-2 toxin [43] and DON [7] exposure on BeWo cells after 6 and 12 h, respectively; however, according to the results of this study, longer exposure (24 h) to the lower concentrations of these mycotoxins can significantly disrupt barrier integrity. Intercellular junctions play a crucial role in formation and maintenance of epithelial barriers [44]. Exposure to DON or T-2 toxin changes the mRNA and protein expression patterns of junctional proteins in BeWo cells. An upregulation of the mRNA expression of OCLD and ZO-1 by lower concentrations of DON and T-2 toxin was observed, while the mRNA expression of E-cadherin was significantly reduced by all concentrations of DON (Table 1). The increase in mRNA levels of ZO-1 was associated with the reduction in protein expression of ZO-1 and indicates a compensatory response to the stress induced by non-cytotoxic levels of DON and T-2 toxin [45,46]. In addition, due to the involvement of ZO-1 in cadherin based cell–cell adhesion, it can be speculated that the increase in the mRNA expression of ZO-1 might be partly associated with the reduction of E-cadherin in protein levels [47,48]. However, for CLDN-3 and 4, the observed reduction in protein expression was not accompanied by prominent changes in mRNA expression. The underlying mechanism responsible for these changes in expression patterns needs further investigation in order to unravel the differences among mycotoxins. Both DON and T-2 toxin belong to the group of trichothecenes and impose their toxic effect by generating the production of free radicals, inducing lipid peroxidation which leads to the imbalance of the antioxidant status of the cells [49]. Moreover, the mitochondria-related caspase-dependent apoptotic pathway can be involved in DON-induced cytotoxicity [50]. Both DON and T-2 toxin can bind to ribosomes and activate mitogen-activated protein kinases (MAPKs) through the initiation of the ribotoxic stress response and induce protein oxidation and apoptosis [49] which could be a possible mechanism involved in the observed effects on TJ expression by DON and T-2 toxin. In this regard, it has been shown that induction of MAPK activation by DON treatment can modulate the expression of claudin-4 in an intestinal epithelial cell line [51].

Exposure to ZEN had less prominent effects on the mRNA and protein expression of TJs, which may partially explain the unchanged TEER and FITC-D permeability in BeWo cell layer after 24 h exposure. High concentrations of ZEN decreased the mRNA levels of TJs but had no significant effect on their protein expressions (Table 1). Structural and functional differences between ZEN and the other two mycotoxins might be responsible for the difference in toxic effects of these mycotoxins. Zearalenone is a non-steroidal estrogenic mycotoxin [52]. It can interact with estrogenic receptors and disturb the cell cycle progression thereby inhibiting cell proliferation [52]. Longer exposure to ZEN can trigger the differentiation of BeWo cells into syncytiotrophoblast-like cells inducing morphological changes as well as human chorionic gonadotropin (hCG) secretion [15]. Possibly, the induction of differentiation in BeWo cells may partially be responsible for the observed effects of ZEN on TJ expression [53]. 

The integrity of the BeWo cell layer is not only maintained by sufficient expression levels of junctional proteins; their cellular localization and distribution are also of critical importance. Mycotoxins DON and T-2 toxin caused reduction and fragmentation of the junctional network of ZO-1, E-cad, and CLDN-4 (Table 1), which corresponded to the observed reduction in protein expression levels of these junctional proteins. Furthermore, an irregular accumulation of OCLD proteins in cells exposed to DON and T-2 toxin was observed. The effect of ZEN on TJ protein assembly was less pronounced. However, after exposure to the highest concentration of ZEN, aberrant structures and membrane dislocations of CLDN-4 and OCLD were observed. Abnormally localized OCLD protein may explain the increased levels of OCLD mRNA expression as a repair mechanism in response to the lack of OCLD protein in the cell membrane. Our observations indicate that these mycotoxins can disrupt the placental barrier, which, in the in vivo situation, may facilitate the transfer of harmful chemicals and toxins through the placenta. Several in vivo and in vitro studies provide evidence on transfer of mycotoxins from maternal blood into the fetal side, where the unchanged and metabolized forms of these toxins can be detected in fetal samples [7,8,43]. Our results show that this may be due, at least in part, to the impaired junctional protein networks of the trophoblast cell layer. 

The observed adverse effects of the tested mycotoxins on placental epithelial integrity can be due to the fact of either their direct effect on the cells or via induction of inflammatory responses. Human trophoblasts are capable of expressing a number of cytokines, including IL-6, which in non-pathologic conditions play an important role in maintenance of pregnancy [54,55]. However, pathologic conditions can alter the cytokine release pattern and enhance IL-6 release [56]. It has been shown that IL-8 production and IL-6 transcription and secretion were concentration-dependently enhanced in BeWo cells, after IL-1 and tumor necrosis factor (TNF)-α stimulation,; however, transforming growth factor (TGF)-β stimulation caused elevated IL-6 levels without affecting IL-8 production [56]. In the current study, we showed that DON, T-2 toxin, and ZEN increased the production of IL-6 in BeWo cells, even at low concentrations, but they did not induce IL-8 release. These observations suggest that tested mycotoxins may stimulate IL-6 production via pathways similar to TGF-β induction. It has been suggested that interleukins can act as modulators of junctional complexes, including those of the placenta [28], and alter the permeability characteristics of epithelial barriers affecting OCLD, CLDNs, and ZO-1 proteins [57]. The cytokine IL-6 can increase paracellular permeability and change the distribution and morphology of TJ protein assembly in endothelial cell layer [58]. In addition, IL-6 increases TJ permeability of intestinal epithelium by altering the expression of claudin-2 protein via an IL-6Rα-coupled signal transducer and gp130 signaling pathway [59]. It has been shown that untreated BeWo cells can express both IL-6R and gp130 mRNA [60]. Therefore, increased IL-6 production in BeWo cells may account for the detrimental effects of the tested mycotoxins and can be partially responsible for the observed irregular localization of the junctional proteins. Apart from its effect on TJs, IL-6 can impose adverse effects on placental transportation through other pathways as well. High levels of IL-6 in maternal blood can stimulate trophoblast fatty acid accumulation and, consequently, contribute to an excessive nutrient transfer across the maternal–fetal barrier [61]. In addition, IL-6 was shown to be transferred to the fetus both in mid and late gestation after intravenous administration in pregnant rats [62]. Prenatal exposure to IL-6 in early and late pregnancy can lead to long-term adverse effects including insulin resistance, elevated stress response, hypertension, and dysregulation of hypothalamic–pituitary–adrenal axis activity during adulthood [63,64].

In this study, IL-6 mRNA expression levels were concentration-dependently increased by exposure to DON and T-2 toxin, but not by ZEN. As mentioned earlier, ZEN may trigger BeWo cell trophoblast differentiation [15] which is accompanied by a marked decrease in mRNA expression of some interleukins including Il-6 [65]. 

In conclusion, our study is the first to report that major *Fusarium* mycotoxins can increase paracellular permeability in BeWo cells by inducing significant changes in expression and delocalization of junctional proteins. Furthermore, the production of IL-6 increased in response to mycotoxin exposure, which can induce detrimental effects on fetal development and might be partially responsible for the mechanisms involved in altered structures of junctional networks. Although ZEN did not induce any obvious effects on barrier function, it significantly increased the IL-6 production, suggesting that mechanisms other than those involving IL-6 might be responsible for observed mycotoxin-induced damages (Table 1). Therefore, further investigations are required to understand the exact mechanism underlying the toxicity of these mycotoxins on the placental barrier. In this study, the effects of direct exposure of major *Fusarium* mycotoxins to the placenta epithelial cell line were investigated and, as we observed from significant changes in BeWo monolayer permeability, inflammatory responses, and structural changes in the cells, it can be inferred that mycotoxins may bring about detrimental consequences on fetal health and cause an imbalanced transportation of nutrients; therefore, the results from our study emphasize the necessity to further investigate the potential detrimental consequences on fetal health. 

## 4. Materials and Methods

### 4.1. Mycotoxins

Purified DON, ZEN, and T-2 toxin (D0156, Z2125, T4887, Sigma–Aldrich, St Louis, MO, USA) were dissolved in absolute ethanol, methanol, and chloroform, respectively, to prepare stock solutions according to the manufacturer’s instructions and were stored at −20 °C. The stock concentrations of DON and ZEN were 32 mM and T-2 toxin was 4 mM. Serial dilutions were prepared in cell culture medium prior to each experiment. 

Different concentrations of DON (1, 2, 4, 8, and 16 µM), ZEN (1, 2, 4, 8 and 16 µM), and T-2 toxin (1, 2, 4, 8, 16, and 32 nM) were used in the assays described below. An incubation time of 24 h was used throughout the experiments related to the daily exposure to these regular food contaminants in an in vivo situation. 

### 4.2. Cell Culture

The human placenta choriocarcinoma (BeWo) cell line was obtained from the American Type Culture Collection (ATCC-CCL-98, Rockville, MD, USA; passages 11–45) and was cultured in culture flasks (Falcon, VWR International, Strasbourg, France) in F-12K medium (Kaighn’s modification of Ham’s F-12 medium; Gibco, Thermo Fisher Scientific, Wilmington, DE, USA), supplemented with 10% fetal bovine serum (Perbio Science, Brebières, France), and 1% penicillin (100 U/ml) and streptomycin (100 µg/ml) mixture. Cells were maintained in a humidified atmosphere of air containing 5% CO_2_ at 37 °C. Medium was refreshed three times per week and confluent cell cultures (7 ± 1 days) were passaged using 0.05% trypsin (Gibco, Thermo Fisher Scientific, Wilmington, DE, USA) and 0.54 mM ethylene diamine tetra-acetic acid (EDTA). 

For mycotoxin exposure, BeWo cells were seeded in 96 or 24 well plates (Falcon, VWR International, Strasbourg, France) at cell densities of 10,000 and 30,000 cell per well, respectively. Once achieving 80% confluency (after 7 days), the cells were exposed to one of the abovementioned mycotoxins for 24 h.

### 4.3. Cell Viability Assay 

Cytotoxic effects of different concentrations of DON (1, 2, 4, 8, and 16 µM), ZEN (1, 2, 4, 8, and 16 µM), and T-2 toxin (1, 2, 4, 8, 16, and 32 nM) on BeWo cells were determined by measuring LDH release from lysed and dead cells in cell culture medium using the CytoTox 96^®^ Non-Radioactive CytoToxicity Assay Kit (Promega Corporation, Madison, WI, USA) according to the manufacturer’s instructions. Cytotoxicity (%) was calculated using the formula: Cytotoxicity (%) = (E/M) × 100%, where E is the experimental release of LDH and M is the maximal release of LDH determined by incubating the cells with a standard lysis solution included in the assay kit. A wide range of mycotoxin concentrations were initially used to make a concentration-response assessment and determine the minimum concentrations of the mycotoxins which can impose toxic effects on the placental epithelial barrier. These mycotoxin concentrations are also related to the concentrations used in other cell-based assays [7,16,43]. In subsequent experiments, mycotoxins were used at concentrations equal to or below the minimum concentration, which caused a significant cytotoxic effect and cell death.

### 4.4. TEER Measurement and Paracellular Tracer Flux Assay

BeWo cells were seeded on high-pore-density polyethylene terephthalate membrane Transwell inserts with 0.4 μm pores and a surface area of 0.3 cm^2^ (Falcon, BD Biosciences, Franklin Lakes, NJ, USA) and placed in a 24 well plate at a cell density of 30,000 cells per Transwell insert. The integrity of the cellular monolayer was evaluated by measuring transepithelial electrical resistance (TEER) using a Millicell-ERS volt-ohm meter (Millipore, Temecula, CA, USA). After 7 days, mean TEER values for untreated confluent cell monolayers were 60 ± 0.6 Ω · cm^2^, as also observed in other BeWo cell cultures [7,23,66,67]. The increasing concentrations of DON, T-2 toxin or ZEN were added to the apical side of the cells and TEER was measured 24 h after mycotoxin exposure. Thereafter, membrane-impermeable FITC-D (molecular mass of 4 kDa, 16 μg/mL; Sigma–Aldrich, St Louis, MO, USA) was added to the apical compartment for 3 h, and paracellular flux was determined by measuring the fluorescence intensity in the basolateral compartment with a fluorometer (Fluoroskan Ascent FL; Thermo Labsystems, Waltham, MA, USA) set at excitation and emission wavelengths of 485 nm and 520 nm.

### 4.5. RNA Extraction and Quantitative qPCR

The BeWo cells, seeded in 24 well plates as described above, were exposed to different mycotoxins for 24 h. Cells were harvested into 100 μL RNA lysis buffer with β-mercaptoethanol (provided within RNA isolation kit) and total RNA was isolated using spin columns based on the manufacturer’s instructions (SV Total RNA Isolation System, Promega Corporation, Madison, WI, USA). Total RNA content of the samples was measured using the NanoDrop ND-1000 Spectrophotometer (Thermo Fisher Scientific, Wilmington, DE, USA). The RNA purity of the samples was confirmed by calculating the 260/280 nm and 260/230 nm ratios, and the samples with the ratios between 1.8 to 2 were considered as high purity. Subsequently, the iScript cDNA Synthesis kit (Bio-Rad Laboratories, Hercules, CA, USA) was used to reverse-transcribe the RNA into cDNA, using the T100 thermal cycler (Bio-Rad Laboratories, Hercules, CA, USA). 

For qPCR, the reaction mixture was prepared by adding specific forward and reverse primers and iQSYBR Green Supermix (Bio-Rad Laboratories, Hercules, CA, USA) to the samples, and amplifications were performed according to the manufacturer’s instructions using the CFX96 Touch™ Real-Time PCR Detection System (Bio-Rad Laboratories, Hercules, CA, USA). Selected primers (Table 2) [45] were commercially manufactured (Biolegio, Nijmegen, Netherlands) and for specificity and efficiency confirmation, qPCR with dilution series of pooled cDNA at a temperature gradient of 55 °C to 65 °C was performed to analyze the melting curves and determine the optimum annealing temperature for each primer. The mRNA quantity was calculated relative to the expression of β-actin reference gene. 

### 4.6. Western Blot Analysis

In order to quantify the protein expression of tight junctions in BeWo cells, Western blot analysis was performed. The cells, seeded in 24 well plates, as described above, were exposed to different mycotoxins for 24 h. Cells were lysed and harvested with RIPA Lysis and Extraction Buffer (Thermo Scientific, Rockford, IL, USA) containing protease inhibitors (Roche Applied Science, Penzberg, Germany). Total protein concentration was assessed by a BCA protein assay kit (Thermo Scientific, Rockford, IL, USA). Equal amounts of protein from heat-denaturized samples were separated by electrophoresis (Criterion™ Gel, 4–20% Tris-HCl, Bio-Rad Laboratories, Hercules, CA, USA) and transferred by Trans-Blot Turbo Transfer System (Bio-Rad Laboratories, Hercules, CA, USA) onto Trans-Blot Turbo Midi PVDF Transfer Packs (Bio-Rad Laboratories, Hercules, CA, USA). Membranes were blocked in blocking buffer (0.05% (*v*/*v*) Tween-20 in PBS (PBST) and 5% (*w*/*v*) milk proteins) for 1 h at room temperature. The membranes were incubated overnight (at 4 °C) with primary antibodies of CLDN-3 and 4, ZO-1 (341700, 329400 and 402200, Invitrogen, Carlsbad, CA, USA), OCLD (AB31721, Abcam, Cambridge, UK) and E-cadherin (610182, eBioscience, San Diego, CA, USA) diluted in blocking buffer according to the manufacturer’s instructions. After washing in PBST 3 times, the membranes were incubated with appropriate secondary antibodies (Dako, Glostrup, Denmark) for 2 h at room temperature. Blots were washed in PBST and incubated with ECL reagents mix (Amersham Biosciences, Roosendaal, The Netherlands) and the bands were visualized using ChemiDoc™ XRS+ System (Bio-Rad Laboratories, Hercules, CA, USA) and analyzed using Image Lab software (version 5.2, 2014, Bio-Rad Laboratories, Hercules, CA, USA). Monoclonal rabbit anti-human β-actin antibody (Cell Signaling, Danvers, MA, USA) was used to evaluate the homogeneity of loading and normalize the optical density of the bands. The protein levels were expressed as the mean fold change in relation to the control group. 

### 4.7. Immunofluorescence Staining

Immunofluorescence staining was conducted to determine cellular localization of ZO-1, OCLD, CLDN-1, 3 and 4, and E-cadherin. The BeWo cells were grown on 24 well plates and exposed to different mycotoxins at the apical compartment for 24 h. The cells were fixed with 10% formalin and washed with PBS prior to permeabilization with 0.1% (*v*/*v*) Triton-X-100 solution for 5–10 min. Samples were blocked for 30 min at room temperature using 5% serum diluted in PBS containing 1% BSA. Thereafter, cells were incubated for 2 h at room temperature with primary antibodies against CLDN3, CLDN4, OCLD, and ZO-1 (341700, 329400, 331500 and 402200, Invitrogen, Carlsbad, CA, USA) or E-cadherin (610182, eBiosciences, San Diego, CA, USA) diluted in 1% PBS–BSA solution according to the manufacturer’s instructions. After washing, cells were incubated with Alexa-Fluor conjugated secondary antibodies (Invitrogen, Carlsbad, CA, USA) for 1 h at room temperature. Anti-fading mounting medium containing DAPI (ProLong Gold with DAPI, Life Technologies, Thermo Fisher Scientific, Wilmington, DE, USA) was used for nuclear counterstaining and sealing the wells with a coverslip. Localization of the junctional proteins was visualized using the Microscope Leica TCS SP8 X.

### 4.8. Quantitative Determination of Interleukin-6 (IL-6), IL-8, and IL-1β

The concentration of IL-6 in the cell culture supernatants was determined after 24 h of mycotoxin exposure in BeWo cells, using the commercially available human IL-6, IL-8, and IL-1β enzyme-linked immunosorbent assay (ELISA) kits (Invitrogen, San Diego, CA, USA) following the guidelines provided by the manufacturer. 

### 4.9. Statistical Analysis

Statistical analyses were performed using GraphPad Prism (version 7.04, 2017, GraphPad, La Jolla, CA, USA). Results are expressed as the mean ± SEM and differences among groups were statistically determined using one-way analysis of variance (ANOVA). When significant differences were observed, Bonferroni post-hoc analysis was used to identify significant differences among the means. Differences were considered significant at *p* < 0.05. Different lowercase letters on the bars indicate significant differences among groups, and letters shared in common between or among the groups indicate no significant difference.

## Figures and Tables

**Table 1:** Summary effects of deoxynivalenol (DON), zearalenone (ZEN), and T-2 toxin on occludin (OCLD), zonula occludens protein-1 (ZO-1), claudin (CLDN)-3 and 4, and E-cadherin (E-cad) profiles.

TJ or AJ	mRNA	Protein	IF Staining
**Mycotoxin DON**			
ZO-1	↑	↓	Decreased and irregular assembly
OCLD	↑	−	Increased delocalization and intracellular accumulation
CLDNs	−	↓	Decreased and modified assembly and belt-like structure
E-cad	↓	↓	Decreased and irregular assembly
**Mycotoxin ZEN**			
ZO-1	↓	−	No obvious effect
OCLD	−	−	Increased delocalization and intracellular accumulation
CLDNs	↓	−	Modified assembly and belt-like structure
E-cad	↓	−	No obvious effect
**Mycotoxin T-2 toxin**			
ZO-1	↑	↓	Decreased and irregular assembly
OCLD	↑		Increased delocalization and intracellular accumulation
CLDNs	−	↓	Decreased and modified assembly and belt-like structure
E-cad	↓	↓	Decreased and irregular assembly

**Table 2:** Primer sequences used for qRT-PCR analysis (AT, annealing temperature (°C)).

Genes	Primer Sequence (5′–3′)		References	AT
	Forward	Reverse		
**β-actin**	CTGGAACGGTGAAGGTGACA	AAGGGACTTCCTGTAACAATGCA	NM-001101	63
**Claudin-3**	CTGCTCTGCTGCTCGTGTC	CGTAGTCCTTGCGGTCGTAG	NM-001306	63
**Claudin-4**	GTCTGCCTGCATCTCCTCTGT	CCTCTAAACCCGTCCATCCA	NM-001305	62.5
**E-cadherin**	TGGACCGAGAGAGTTTCCCT	CCCTTGTACGTGGTGGGATT	BC-144283.1	60
**Occludin**	TTGGATAAAGAATTGGATGACT	ACTGCTTGCAATGATTCTTCT	NM-002538	57
**ZO-1**	GAATGATGGTTGGTATGGTGCG	TCAGAAGTGTGTCTACTGTCCG	NT-010194.17	55.8
**IL-6**	TACCCCCAGGAGAAGATTCC	TTTTCTGCCAGTGCCTCTTT	S56892.1	63
**IL-8**	CTCTTGGCAGCCTTCCTGATT	TATGCACTGACATCTAAGTTCTTTAGCA	NM-000584.3	60
**IL-1β**	GCTGAGGAAGATGCTGGTTC	GTGATCGTACAGGTGCATCG	NM-000756	57
